# Ultrasonic and clinical risk factors of solitary Plaque-RADS-2 carotid plaque for predicting anterior circulation ischemic stroke

**DOI:** 10.3389/fcvm.2025.1631110

**Published:** 2025-10-24

**Authors:** Shuangshuang Zhao, Zheng Zhang, Xincai Wu, Yanwei Chen, Yun Cai, Huajiao Zhao, Jiayan Bao, Wenjun Li, Jiwen Qian, Xin Zhang, Baoding Chen

**Affiliations:** Department of Ultrasound Medicine, Affiliated Hospital of Jiangsu University, Zhenjiang, Jiangsu Province, China

**Keywords:** ischemic stroke, carotid plaque, Plaque-RADS-2, risk factor, ultrasound

## Abstract

**Background:**

Plaque-RADS-2 carotid plaque is regarded as a low risk for attribution of cerebrovascular events. Nevertheless, in individuals with carotid plaque, the risk of ischemic stroke poses a continuous threat to both health and quality of life. This study aimed to investigate risk predictors of anterior circulation ischemic stroke (ACIS) in patients with solitary Plaque-RADS-2 carotid plaque.

**Methods:**

Medical records of 1,961 patients were collected at our hospital from January to December 2022. Patients with solitary Plaque-RADS-2 carotid plaque were divided into ACIS and non-ACIS groups based on brain CT/MRI results. Ultrasonic and clinical characteristics were assessed by univariate and multivariate analysis.

**Results:**

A total of 134 patients with solitary Plaque-RADS-2 carotid plaque were enrolled in this study. Systolic blood pressure (SBP) >141.5 mmHg, glycosylated hemoglobin (HbA1c) > 7.05%, triglyceride glucose (TyG) index > 7.18, and mainly hypoechoic significantly were predictive factors for ACIS. The prediction equation was logit (*P*) = 2.087 × (if SBP > 141.5 mmHg) + 2.126 × (if HbA1c > 7.05%) + 1.225 × (if TyG index > 7.18) + 1.554 × (if mainly hypoechoic) − 1.795. The area under curve of receiver operating characteristic curve was 0.843.

**Conclusion:**

This study demonstrated that SBP > 141.5 mmHg, HbA1c > 7.05%, TyG index > 7.18, and mainly hypoechoic were independent predictors of ACIS in patients with solitary Plaque-RADS-2 carotid plaque. Significant attention should be given to these risk factors to improve the management of solitary Plaque-RADS-2 carotid plaque.

## 1 Introduction

Stroke, ranked second globally and first in China, has become a major public health challenge for prevention and control, owing to its high incidence, disability, recurrence, and economic burden (1, 2). Ischemic stroke (IS) has occupied a dominant position due to its rising frequency and high recurrence rate, accounting for 87% and 63% of all stroke subtypes in the USA and worldwide, respectively (3). The Trial of Org 10172 in Acute Stroke Treatment (TOAST) (Standards of Care for Acute Stroke) criteria stated that carotid artery plaques are considered a cause of anterior circulation ischemic stroke (ACIS) only when ipsilateral internal carotid artery stenosis exceeds 50% (4). Non-stenotic carotid artery disease, defined as <50% stenosis of the internal carotid artery lumen, may be the potential reason for IS, but these patients are still categorized as having cryptogenic stroke or embolic stroke of unknown origin (5). Emerging evidence suggests that some features of plaque vulnerability (e.g., plaque ulceration, intraplaque hemorrhage, or increased plaque thickness) may be independent risk factors for inducing cerebrovascular events, regardless of the degree of stenosis (6). In addition, vulnerable plaques are associated with a high risk of IS and the risk of subsequent vascular events in symptomatic patients and asymptomatic individuals in the general population, making the identification of vulnerable plaques critical (7). Thus, the degree of carotid stenosis is no longer the only imaging metric utilized in clinical settings to determine the best course of treatment.

Last year, the American College of Cardiology Foundation recommended Carotid Plaque-RADS to enhance the transparent and organized sharing of plaque data between imaging and referring physicians and researchers (8). They assessed each vessel and plaque using ultrasound (US), computed tomography (CT) angiography, and magnetic resonance imaging (MRI) and classified the plaque into four categories to produce a standardized report. Notably, Plaque-RADS-2 carotid plaque, which is regarded as a low risk for attribution of cerebrovascular events, is defined in this classification as eccentric plaque with a maximum wall thickness (MWT) <3 mm and without complex plaque characteristics (such as intraplaque hemorrhage, fibrous cap rupture, and intraluminal thrombus). Clinical research has shown that a proportion of patients get ACIS, even if carotid plaques were very small (9, 10). This study aims to identify risk factors of ACIS in patients with solitary Carotid Plaque-RADS 2 plaque based on clinical and ultrasonic characteristics, further predict the occurrence of ACIS and optimize the management of this type of plaque.

## 2 Materials and methods

### 2.1 Population and study design

A total of 1,064 stroke patients from Department of Neurology underwent carotid US examination between January and December 2022, whose medical records (brain CT/MR examinations, laboratory tests, etc.) were collected for analysis. The medical records of 897 non-stroke patients from the Department of Neurology for carotid US examination were gathered during the same period. Patients with intracerebral and subarachnoid hemorrhage and non-vascular causes for stroke were excluded based on brain CT/MRI results, and IS was diagnosed in patients with a compatible neurological deficit after 24 h. This retrospective study was approved by the ethics committee of our institution and conducted in accordance with the Declaration of Helsinki. Informed consent was obtained from the patients before the examination.

The inclusion criteria for the ACIS group were as follows: (1) diagnosis of ACIS and (2) presence of a solitary carotid plaque with a MWT of <3 mm. The exclusion criteria were as follows: (1) evidence of cardiogenic stroke; (2) hemorrhagic stroke; (3) history of stroke, transient ischemic attack (TIA), or coronary artery disease (CAD); (4) absence of brain CT/MRI results; and (5) multiple plaques in the carotid arteries or a solitary plaque with a MWT ≥3 mm; and (6) absence of carotid US results.

The inclusion criteria for the non-ACIS group were as follows: (1) presence of a solitary carotid plaque with an MWT of <3 mm; (2) no image features of ACIS by brain CT/MRI examination; and (3) no neurological deficit symptoms related to ACIS. The exclusion criteria were as follows: (1) history of stroke, TIA, or CAD; (2) absence of brain CT/MRI results; (3) patients with multiple plaques in the carotid arteries or a solitary plaque with a MWT ≥ 3 mm; and (4) absence of carotid US results.

Clinical characteristics [gender, age, weight, stature, body mass index (BMI), blood pressure (BP), hypertension, diabetes, smoking, receive BP reduction, glucose-lowering therapy, lipid-lowering therapy, antiplatelet therapy, etc.], laboratory results [fasting blood glucose (FBG), glycosylated hemoglobin (HbA1c), triglycerides, total cholesterol, high-density lipoprotein cholesterol (HDL-C), and low-density lipoprotein cholesterol (LDL-C) levels, apolipoprotein A-I and apolipoprotein B], and brain CT/MRI results were obtained from electronic medical records. Patients were divided into the ACIS group and the non-ACIS group according to CT/MRI brain results.

The BP values used for data analysis in this study were the average of two BP measurements at the survey site. Hypertension was defined when any of the two following conditions were satisfied: (1) systolic blood pressure (SBP) ≥ 140 mmHg and/or diastolic blood pressure (DBP) ≥ 90 mmHg and (2) history of hypertension or taking BP reduction medication within 2 weeks. Diabetes was diagnosed based on the following: FBG ≥ 7.0 mmol/L, a 2 h serum glucose level ≥ 11.1 mmol/L on oral glucose tolerance testing, or the current use of glucose-lowering drugs or insulin. BMI was calculated by dividing weight by height squared. Triglyceride glucose (TyG) index = Ln [fasting triglycerides (mg/dL)  × FBG (mg/dL)/2] (11).

### 2.2 Conventional US examinations

Sonograms for the carotid artery were performed with an Esaote MyLab Twice color Doppler US diagnostic machine (LA523-type high frequency linear array, frequency range: 5–18 MHz) by two radiologists with 5–8 years ' experience.

In a supine position, with the head slightly inclined to the contralateral side, a normal US scan was performed on each patient, encompassing the complete viewable length of the extrathoracic common carotid artery, carotid bifurcation, and extracranial internal carotid artery and external carotid artery on both sides. Conventional US characteristics including carotid intima–media thickness (IMT) and resistance index (RI) of common carotid artery on both sides, and the thickness, length, location, echogenicity and calcification of solitary plaque were measured and recorded. Plaque is identified by US when carotid IMT is larger than 1.5 mm or is convex to carotid artery lumen and measures 0.5 mm or >50% of surrounding IMT (12). Calcification types of plaques are punctate (<1 mm in diameter) and fragmented (>1 mm in diameter) (13).

### 2.3 Statistical analysis

Continuous variables were expressed as mean ± standard deviation (SD). Comparisons of clinical and sonographic characteristics between groups were performed using Student's *t*-test, Pearson's *χ*^2^ test, or Fisher's exact test. Multifactorial logistic regression analysis was performed to determine risk factors for predicting ACIS in patients with solitary Plaque-RADS-2 carotid plaque and to derive logistic regression equations. Receiver operating characteristic (ROC) curve was used to calculate cutoff values, area under the curve (AUC), sensitivity and specificity values, and Youden index. *P* < 0.05 was considered statistically significant. All statistical analyses were performed using the SPSS 26.0 statistical package (SPSS, Inc., Chicago, IL, USA).

## 3 Results

### 3.1 Clinical characteristics of the study population

**Figure 1:**
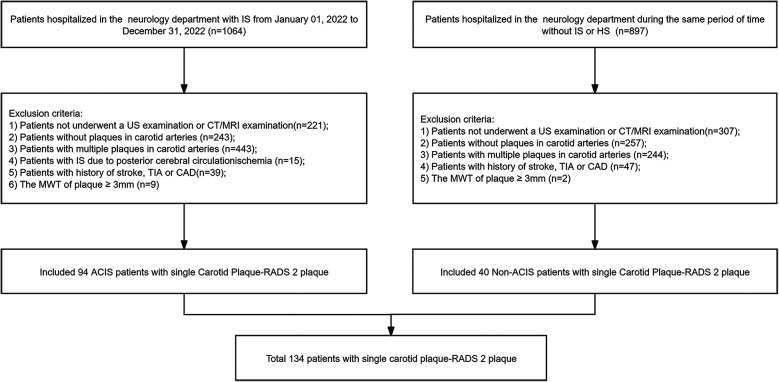
Flowchart of this study. IS, ischemic stroke; HS, hemorrhagic stroke; US, ultrasound; CT, computed tomography; MRI, magnetic resonance imaging; TIA, transient ischemic attack; CAD, coronary artery disease; MWT, maximum wall thickness; ACIS, anterior circulation ischemic stroke.

In this study, medical records of 1,064 patients diagnosed with stroke were collected from the Department of Neurology at our hospital between January and December 2022. Moreover, medical records of 897 non-stroke patients were collected during the same period. As shown in Figure 1, a total of 134 patients met the inclusion criteria and were incorporated into the study. This cohort included 69 females (mean age, 68.36 ± 10.99 years; age range, 40–93 years) and 65 males (mean age, 64.95 ± 10.63 years; age range, 34–91 years). Based on brain CT/MRI results, 94 patients were assigned to ACIS group, and the remaining patients were assigned to non-ACIS group. Clinical baseline and biochemical characteristics are presented in Table 1. Weight, SBP, DBP, FBG, HbA1c, triglycerides, TyG index, and hypertension were associated with ACIS in patients with solitary Plaque-RADS-2 carotid plaque (all *P* < 0.05).

**Table 1:** Baseline clinical and biochemical characteristics in 134 individuals with solitary Plaque-RADS-2 carotid plaque.

Characteristics	Non-ACIS (*n* = 40)	ACIS (*n* = 94)	*P*-value
Gender (%)			0.822
Female	20 (50.0)	49 (52.1)	
Male	20 (50.0)	45 (47.9)	
Age (year)	66.80 ± 10.94	66.67 ± 10.95	0.950
Weight (kg)	62.00 ± 11.45	67.05 ± 11.54	0.022
Stature (m)	1.62 ± 0.09	1.64 ± 0.08	0.100
BMI (kg/m^2^)	23.61 ± 3.87	24.76 ± 3.41	0.091
SBP (mmHg)	135 ± 20	152 ± 19	<0.001
DBP (mmHg)	76 ± 13	85 ± 14	0.002
FBG (mmol/L)	5.85 ± 1.93	6.97 ± 2.71	0.008
HbA1c (%)	6.18 ± 0.95	7.04 ± 2.02	0.001
Triglycerides (mmol/L)	1.37 ± 0.64	1.99 ± 1.75	0.004
Total cholesterol (mmol/L)	4.46 ± 1.09	4.65 ± 1.12	0.367
HDL-C (mmol/L)	1.12 ± 0.40	1.04 ± 0.28	0.172
LDL-C (mmol/L)	2.56 ± 0.92	2.60 ± 0.89	0.786
Apolipoprotein A-I (g/L)	1.15 ± 0.26	1.09 ± 0.17	0.252
Apolipoprotein B (g/L)	0.81 ± 0.24	0.83 ± 0.26	0.698
TyG index	7.05 ± 0.48	7.44 ± 0.77	<0.001
Hypertension, *n* (%)			<0.001
No	14 (35.0)	10 (10.6)	
Yes	26 (65.0)	84 (89.4)	
Diabetes, *n* (%)			0.051
No	32 (80.0)	59 (62.8)	
Yes	8 (20.0)	35 (37.2)	
Smoking, *n* (%)			0.567
No	28 (70.0)	61 (64.9)	
Yes	12 (30.0)	33 (35.1)	
Blood pressure reduction, *n* (%)			0.128
No	21 (52.5)	36 (38.3)	
Yes	19 (47.5)	58 (61.7)	
Glucose-lowering therapy, *n* (%)			0.528
No	33 (82.5)	73 (77.7)	
Yes	7 (17.5)	21 (22.3)	
Lipid-lowering therapy, *n* (%)			0.317
No	40 (100)	90 (95.7)	
Yes	0 (0.0)	4 (4.3)	
Antiplatelet therapy, *n* (%)			0.669
No	39 (97.5)	89 (94.7)	
Yes	1 (2.5)	5 (5.3)	

ACIS, anterior circulation ischemic stroke; BMI, body mass index; SBP, systolic blood pressure; DBP, diastolic blood pressure; FBG, fasting blood glucose; HbA1c, glycosylated hemoglobin; HDL-C, high-density lipoprotein cholesterol; LDL-C, low-density lipoprotein cholesterol; TyG, triglyceride glucose.

### 3.2 Conventional US characteristics of solitary Plaque-RADS-2 carotid plaque

**Figure 2:**
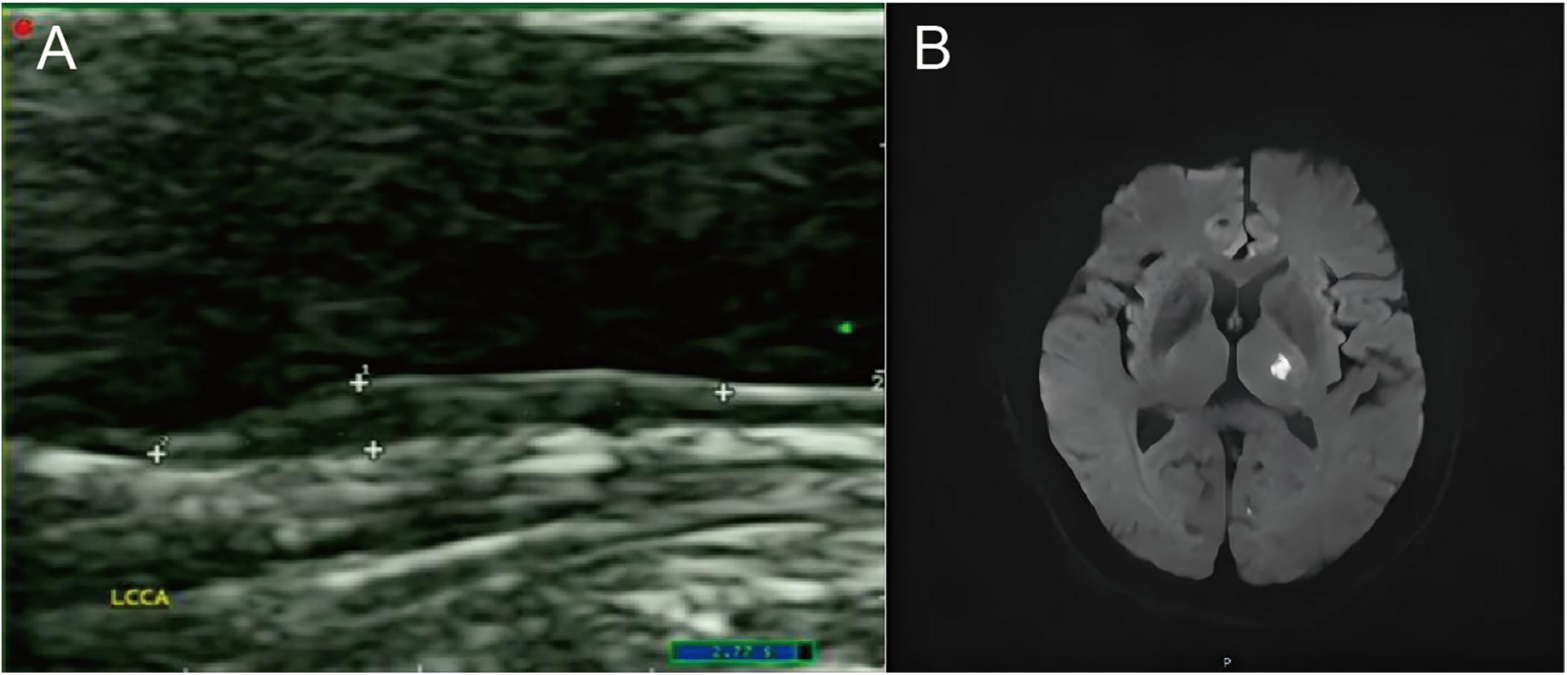
A case of an ACIS patient with a solitary Plaque-RADS-2 carotid plaque. (**A**) The sonogram shows a mainly hypoechoic plaque on the posterior wall of the left common carotid artery. (**B**) Diffusion-weighted imaging (DWI) sequence shows high signal in the left basal ganglia.

Table 2 lists the carotid-related measurements and US characteristics of plaques in ACIS and non-ACIS groups. Results revealed that among US characteristics of solitary Plaque-RADS-2 carotid plaques, only mainly hypoechoic characteristic was associated with ACIS (*P* < 0.05) (Figure 2).

**Table 2:** Ultrasonic characteristics in 134 individuals with solitary Plaque-RADS-2 carotid plaque.

Characteristics	Non-ACIS (*n* = 40)	ACIS (*n* = 94)	*P*-value
Right IMT	0.81 ± 0.14	0.78 ± 0.14	0.327
Left IMT	0.81 ± 0.15	0.81 ± 0.14	0.886
Right RI	0.73 ± 0.07	0.74 ± 0.07	0.861
Left RI	0.72 ± 0.06	0.73 ± 0.06	0.342
Plaque thickness, mm	1.89 ± 0.32	2.01 ± 0.39	0.085
Plaque length, mm	7.65 ± 3.82	7.84 ± 3.81	0.797
Plaque location, *n* (%)			0.735
Left carotid artery	20 (50.0)	44 (46.8)	
Right carotid artery	20 (50.0)	50 (53.2)	
Plaque position, *n* (%)			0.282
Anterior	5 (12.5)	18 (19.2)	
Lateral	0 (0.0)	2 (2.1)	
Posterior	35 (87.5)	74 (78.7)	
Mainly hypoechoic, *n* (%)			0.006
No	27 (67.5)	39 (41.5)	
Yes	13 (32.5)	55 (58.5)	
Thickness of fibrous cap			0.554
Thick	40 (100.0)	91 (96.8)	
Thin	0 (0.0)	3 (3.2)	
Number of calcifications, *n* (%)			0.134
None	20 (50.0)	50 (53.2)	
Solitary	4 (10.0)	20 (21.3)	
Multiple	16 (40.0)	24 (25.5)	
Classification of calcifications, *n* (%)			0.751
None	20 (50.0)	50 (53.2)	
Punctate	14 (35.0)	27 (28.7)	
Fragmented	6 (15.0)	17 (18.1)	

ACIS, anterior circulation ischemic stroke; IMT, intima–media thickness; RI, resistance index.

### 3.3 Multifactorial logistic regression analysis for predicting ACIS

**Figure 3:**
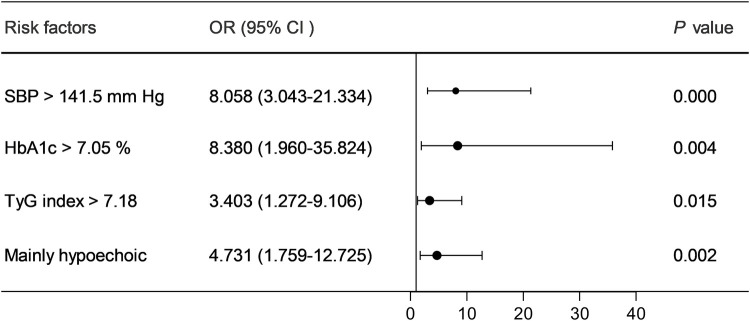
Forest plot of the risk factors of ACIS in patients with solitary Plaque-RADS-2 carotid plaque.

**Figure 4:**
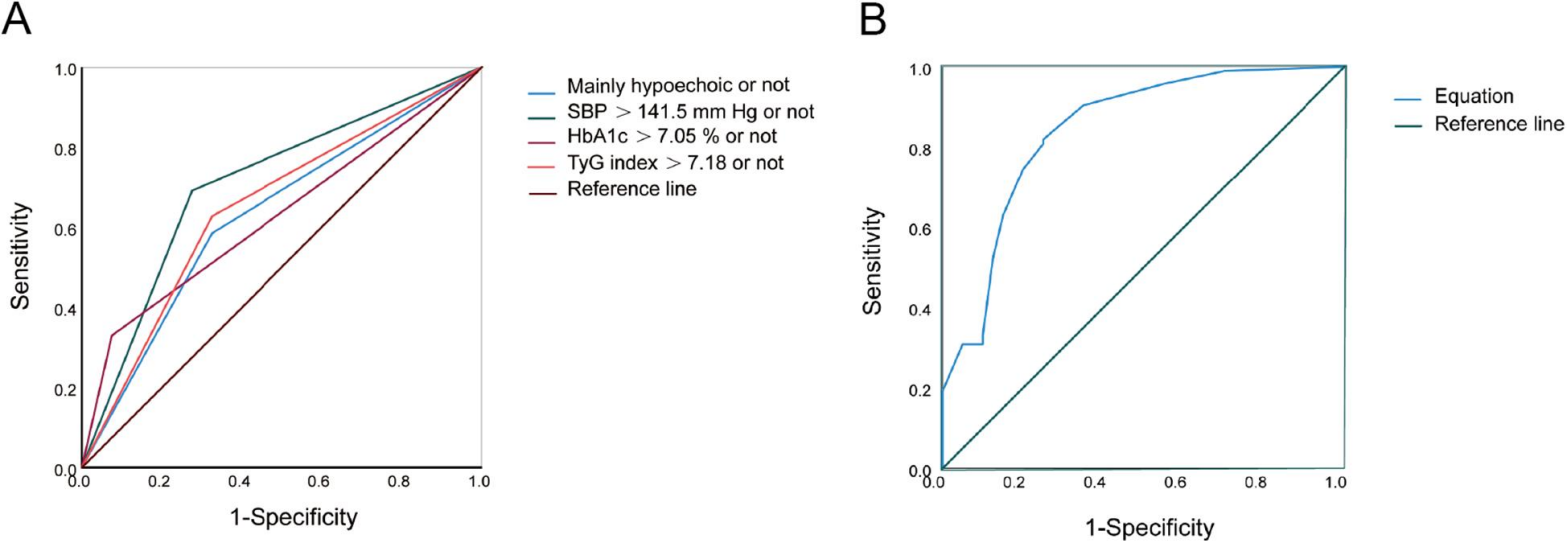
ROC curves of risk factors and equation for predicting ACIS in patients with solitary Plaque-RADS-2 carotid plaque. (**A**) Mainly hypoechoic [area under the ROC curve (AUROC) = 0.630], SBP >141.5 mmHg (AUROC = 0.731), HbA1c >7.05% (AUROC = 0.611), and TyG index >7.18 (AUROC = 0.672), respectively. (**B**) Equation (AUROC = 0.843) for predicting ACIS in patients with solitary Plaque-RADS-2 carotid plaque (all *P* < 0.05).

Multifactorial logistic regression analysis showed that SBP > 141.5 mmHg [odds ratio (OR) = 8.058; 95% confidence interval (CI) = 3.043–21.334; *P* = 0.000], HbA1c > 7.05% (OR = 8.380; 95% CI = 1.960–35.824; *P* = 0.004), TyG index > 7.18 (OR = 3.403; 95% CI = 1.272–9.106; *P* = 0.015), and mainly hypoechoic (OR = 4.731; 95% CI = 1.759–12.725; *P* = 0.002) were risk factors for ACIS patients with solitary Plaque-RADS-2 carotid plaque (Figure 3, Table 3). Hosmer–Lemeshow goodness-of-fit test result yielded *P* = 0.116, indicating that the model fit is good. The AUCs of these factors' ROC curves were 0.731 (95% CI = 0.634–0.828), 0.611 (95% CI = 0.513–0.710), 0.672 (95% CI = 0.578–0.767), and 0.630 (95% CI = 0.528–0.733), respectively. In addition, their sensitivity and specificity values were 69.1% and 72.5%, 33.0% and 92.5%, 64.9% and 67.5%, and 58.5% and 32.5%, respectively (Figure 4A and Table 4).

**Table 3:** Multivariate logistic regression analysis for predicting ACIS in patients with solitary Plaque-RADS-2 carotid plaque.

Risk factors	*β* Coefficient	OR	95% CI	*P*-value
SBP >141.5 mmHg	2.087	8.058	3.043–21.334	0.000
HbA1c >7.05%	2.126	8.380	1.960–35.824	0.004
TyG index >7.18	1.225	3.403	1.272–9.106	0.015
Mainly hypoechoic	1.554	4.731	1.759–12.725	0.002

ACIS, anterior circulation ischemic stroke; CI, confidence interval; SBP, systolic blood pressure; HbA1c, glycosylated hemoglobin; TyG, triglyceride glucose.

**Table 4:** ROC analysis of the independent factors and equation for predicting ACIS in patients with solitary Plaque-RADS-2 carotid plaque.

Risk factors	AUC	95% CI	Cut off value	Sensitivity	Specificity
SBP >141.5 mmHg	0.731	0.634–0.828	SBP >141.5 mmHg	0.691	0.725
HbA1c >7.05%	0.611	0.513–0.710	HbA1c >7.05%	0.330	0.925
TyG index >7.18	0.672	0.578–0.767	TyG index >7.18	0.649	0.675
Mainly hypoechoic	0.630	0.528–0.733	Mainly hypoechoic	0.585	0.325
Predictive equation	0.843	0.765–0.920	0.58	0.819	0.750

ROC, receiver operating characteristic; ACIS, anterior circulation ischemic stroke; AUC, area under the curve; CI, confidence interval; SBP, systolic blood pressure; HbA1c, glycosylated hemoglobin; TyG, triglyceride glucose.

A binary logistic regression equation was established using these variables: logit(P)=2.087×(ifSBP>141.5mmHg)+2.126×(if HbA1c > 7.05%)  +1.225×(ifTyGindex>7.18)+1.554×(if mainlyhypoechoic)−1.795. AUC of the binary logistic regression equation's ROC was 0.843 (*P* = 0.000, 95% CI = 0.765–0.920). Youden index, sensitivity, and specificity were 0.569, 81.9% and 75.0%, respectively (Figure 4B).

## 4 Discussion

Carotid atherosclerosis, a manifestation of systemic atherosclerosis, is an independent risk factor for stroke and can be detected non-invasively. Mild-to-moderate carotid artery stenosis may be asymptomatic, but if left unchecked, it can lead to severe stenosis or occlusion and cause stroke. The results of this study showed that approximately 56.39% (600/1,064) of stroke patients had varying degrees of carotid plaque and approximately15.70% (167/1,064) had a solitary carotid plaque. Although the attributable risk of cerebrovascular events to Plaque-RADS-2 carotid plaque is low according to the latest Carotid Plaque-RADS classification, the results of present study showed that approximately158.83% (94/1,064) of patients with solitary Plaque-RADS-2 carotid plaque among all stroke patients suffered ACIS. Multifactorial logistic regression analysis revealed that SBP > 141.5 mmHg, HbA1c > 7.05%, TyG index > 7.18, and mainly hypoechoic were risk predictors of ACIS. Logistic regression equation based on these predictors was established, and AUC of ROC curve was 0.843 (*P* = 0.000).

Hypertension is an important metabolic disease and a major risk factor for stroke and cardiovascular disease. Although the solitary Plaque-RADS-2 carotid plaque had no intraplaque hemorrhage, fibrous cap rupture, or intraluminal thrombus, as well as MWT < 3 mm, results of this study still showed that hypertension was associated with ACIS. Moreover, SBP > 141.5 mmHg was the optimal cutoff value for predicting ACIS. The management of BP in acute IS remains a subject of controversy. Wang et al. (14) explored a cohort of 4,069 patients with acute IS from 26 hospitals. Five systolic BP trajectories were identified by using latent mixture modeling to seek the relationship between 24 h BP patterns following IS and clinical outcomes. They found that patients with trajectory category 5 (190–170 mmHg) exhibited the highest risk, while those with trajectory category 1 (150–130 mmHg) had the lowest risk of adverse outcomes at 3-month follow-up. Thus, many randomized controlled trials compared clinical outcomes of early antihypertensive treatment with no antihypertensive treatment or intensive antihypertensive treatment and standard antihypertensive treatment in patients with acute stroke. A meta-analysis of 13 randomized controlled trials, which included 12,703 patients with acute IS, showed that antihypertensive treatment within 3 days of symptom onset did not affect the risk of functional dependence or death at 90 days (15). A multicenter, randomized, open-label trial enrolled 4,810 patients from 106 Chinese hospitals with acute IS within 24–48 h of symptom onset and elevated systolic BP between 140 and 220 mmHg. They found that among patients with mild-to-moderate acute IS and systolic BP between 140 and 220 mmHg who did not receive intravenous thrombolytic treatment, early antihypertensive treatment did not reduce the odds of dependency or death at 90 days (16). Although many mechanisms may contribute to elevated BP in the acute phase of IS, most patients have a previous history of hypertension. Therefore, for patients with solitary Plaque-RADS-2 carotid plaque, actively controlling BP is still required to prevent IS.

HbA1c is a crucial indicator of chronic blood glucose levels, reflecting the average blood glucose level in the 2–3 months before the test, and is unaffected by fluctuations in blood glucose brought on by acute stress (17). Hyperglycemia on admission is more common in acute cerebral infarction and is a contributing factor to the poor prognosis of cerebral infarction (18). One study categorized patients into three groups based on baseline HbA1c, and the findings demonstrated that HbA1c level was an independent predictor of a worse functional prognosis in individuals with acute ACIS. Furthermore, high levels of HbA1c were associated with early neurological deterioration in acute IS patients, and this was better than FBG in early neurological deterioration prediction (19). Xu et al. (20) discovered that there was a positive correlation between HbA1c and poststroke cognitive impairment when it was higher than 8.2%. Our study showed that HbA1c > 7.05% is an independent predictor of ACIS in patients with solitary Plaque-RADS-2 carotid plaque; therefore, active glucose management should be considered in patients with this plaque type to prevent the occurrence of ACIS.

TyG index, which combines fasting glucose and triglyceride levels, serves as a reliable marker for insulin resistance and has been increasingly recognized for its role in predicting cardiovascular and cerebrovascular events. TyG index accelerates the instability of atherosclerotic plaques, contributes to death in patients with metabolic syndrome through innate immunoreactive substances and systemic inflammatory reactive substances (21), and promotes the production of advanced glycosylation end-products, which lead to arterial vascular stiffness (22). Long-term studies, such as an 11-year community-based follow-up in China, have demonstrated that higher TyG index levels independently predict the occurrence of IS (23). This suggested that TyG index could be used as a prognostic tool for identifying individuals at higher risk of stroke. A meta-analysis of 18 studies pooled effect values of all stroke types showed that a higher TyG index was associated with an increased risk of IS in the general population. In addition, IS patients with a higher TyG index were associated with a higher risk of stroke recurrence and increased risk of mortality (24). This highlights the importance of managing TyG index levels in secondary stroke prevention. TyG index is also linked to the instability of atherosclerotic plaques and an increased plaque burden in vulnerable plaques. This instability can lead to plaque rupture, a common cause of acute ischemic events such as stroke (22, 25). This suggests that TyG index can be used for the prevention of atherosclerosis in IS patients. As mentioned above, a higher TyG index is associated with the development of atherosclerosis, and atherosclerosis, as the most important pathogenesis of IS, further influences the development of stroke. A 9-year prospective dynamic study demonstrated that long-term exposure to elevated TyG index levels significantly contributes to stroke risk, emphasizing the need for early and sustained intervention (26). Further study could focus on developing standardized guidelines for the use of TyG index in clinical practice and exploring targeted therapies to manage elevated TyG index levels effectively for prevention of IS.

Although digital subtraction angiography (DSA) is the gold standard for diagnosis of carotid stenosis, the assessment of plaque stability is comparatively poor. CT angiography and MR angiography can provide a reliable assessment of plaque stability, but they are costly and invasive. Carotid US examination has become widely used throughout the world, due to its simplicity, reproducibility, and noninvasiveness. The presence of a thin fibrous cap, hypoechoic plaque, or ulcerated plaque indicates vulnerable plaques in the carotid arteries, which are also known etiological factors contributing to cerebrovascular events. When vulnerable atherosclerotic plaques rupture and plaque material and thrombus embolize into distal arteries, resulting in stroke (27, 28). As with previous findings, hypoechoic plaque is a risk predictor for the development of IS (10). Our results suggested that mainly hypoechoic was a risk factor for predicting ACIS in patients with solitary Plaque-RADS-2 carotid plaque. That is to say, even if the solitary plaque's MWT was <3 mm without complex plaque features (i.e., absence of intraplaque hemorrhage, fibrous cap rupture, and intraluminal thrombosis), a mainly hypoechoic plaque was still a risk factor for the development of IS. In this study, hypoechoic plaque was most likely to consist of a lipid-rich necrotic core. The results of a systematic review and meta-analysis showed that, similar to intraplaque hemorrhage and calcification plaques, lipid-rich necrotic core plaque was associated with stroke as well (29). The probability of IS was 8.83% (94/1,064) in this study. Although Plaque-RADS-2 carotid plaques were considered as low-risk plaques, close attention is still required in clinical practice.

The study has several limitations. First, identification of carotid plaque was performed by US in this study. Although DSA is the gold standard for diagnosis of carotid stenosis, carotid artery stenosis corresponding to plaques included in this study was <50%. For this type of plaque, US remains the primary modality for plaque evaluation and follow-up. In addition, sonograms of carotid artery were performed by two radiologists with 5–8 years' experience in our study. Second, contrast-enhanced ultrasound characteristics of Plaque-RADS-2 carotid plaques were not evaluated in this study. Further study can be considered for predicting ACIS in patients with Plaque-RADS-2 carotid plaques using contrast-enhanced ultrasound characteristics. Third, we only investigated the solitary Plaque-RADS-2 carotid plaque. Multiple plaques were not evaluated since they may have an impact on the associations between certain plaque features and ACIS and an influence on the borderline significance of the presented results. Finally, the sample of this study was small, and non-stroke patients were selected from the Department of Neurology which may introduce selection bias and not accurately represent general population without ACIS. A larger sample of individuals with Plaque-RADS-2 plaques can increase the generalizability in future studies.

## 5 Conclusion

In conclusion, SBP > 141.5 mmHg, HbA1c > 7.05%, TyG index > 7.18, or mainly hypoechoic provides the prediction of future ACIS in patients with solitary Plaque-RADS-2 carotid plaque. Patients with this type of plaque need to manage well their SBP, HbA1c, and TyG index if the echogenicity of the plaque is predominantly hypoechoic.

## Data Availability

The raw data supporting the conclusions of this article will be made available by the authors, without undue reservation.
